# Post‐Stroke Limitations on Activities of Daily Living and Associated Factors in Public Tertiary Hospitals in Amhara Regional State, Ethiopia: A Multicenter Cross‐Sectional Study

**DOI:** 10.1002/hsr2.71884

**Published:** 2026-02-18

**Authors:** Melese Gobezie, Getachew Azeze Eriku, Tesfa Kassa, Destaw Marie Merawie, Samual Mersha Birru, Setegn Fentahun, Gerum Nakie, Jemal Suliuman, Mulualem Kelebie, Alemu Kassaw Kibret

**Affiliations:** ^1^ Department of Physiotherapy, School of Medicine University of Gondar Gondar Ethiopia; ^2^ Department of Nurse, School of Nursing, College of Medicine and Health Sciences University of Gondar Gondar Ethiopia; ^3^ Department of Psychiatry, School of Medicine, College of Medicine and Health Sciences University of Gondar Gondar Ethiopia

**Keywords:** activities of daily living, Amhara regional state, functional disability, older adults, prevalence

## Abstract

**Background:**

Stroke survivors often face challenges with basic activities of daily living (ADLs), which can compromise their health by making it difficult to perform everyday tasks independently. These limitations frequently lead to sedentariness, increased morbidity, and mortality. Although the prevalence of post‐stroke limitations in basic ADLs varies significantly among studies, there is a general lack of data, particularly from Ethiopia. This lack of research hinders a comprehensive understanding of the extent of ADL limitations among stroke survivors in the region and their associated health outcomes.

**Aims:**

To assess the extent of post‐stroke limitation in basic ADL and identify associated factors among stroke survivors.

**Methods:**

An institution‐based cross‐sectional study was conducted from April 1 to June 30, 2022. We selected 400 participants using systematic random sampling. The Barthel Index was used to assess limitations on ADL post‐stroke participants. We checked for multicollinearity and model fitness. Variables with a *p*‐value below 0.25 in bivariable regression were included in a multivariable logistic regression analysis, considering a *p*‐value below 0.05 as statistically significant.

**Results:**

The overall prevalence of post‐stroke limitations in basic ADL was 58.25%. Significant associations were found with symptoms of depression (odds ratio [OR] = 1.69, 95% confidence interval [CI] = 0.88–2.49; *p* < 0.05), right hemisphere stroke (OR = 1.79, 95% CI = 3.58–5.01; *p* < 0.05), absence of early physiotherapy (OR = 1.88, 95% CI = 0.19–1.58; *p* < 0.05), and use of mobility aids (OR = 1.81, 95% CI = 0.17–1.44; *p* < 0.05).

**Conclusion:**

The study highlights the high prevalence of limitations in basic ADL following a stroke, with significant associations found with factors such as stroke in the right hemisphere, lack of early physiotherapy, use of mobility aids, and depressive symptoms. These findings emphasize the need for early rehabilitation interventions, particularly physiotherapy, and addressing mental health to improve recovery outcomes. Healthcare providers should focus on individualized care plans that include physical and psychological support to better manage post‐stroke limitations and enhance the quality of life for stroke survivors.

## 1 Introduction

Stroke is defined by the World Health Organization (WHO) as the rapid development of clinical signs of focal (or global) disturbance in cerebral function, persisting for over 24 h or leading to death, with no apparent cause other than vascular origin [1]. It remains the leading cause of disability and mortality globally, particularly in low‐ and middle‐income countries (LMICs) [2, 3, 4]. Worldwide, of the 50 million people who survive a stroke, between 25% and 74% will face physical, cognitive, and emotional challenges, many of whom will become dependent on activities of daily living (ADLs) [5]. ADL limitations refer to performing fundamental daily activities such as bathing, dressing, eating, and mobility, which are key determinants of quality of life [6].

The impact of stroke is particularly strong in Sub‐Saharan Africa (SSA), where the incidence of stroke is on the rise, and the region is experiencing an escalating burden of non‐communicable diseases [7]. In SSA, stroke rates range 100 to 300 per 100,000 population per year, with mortality and disability rates significantly higher than in high‐income countries due to limited access to care and rehabilitation services [7, 8]. Within Ethiopia, stroke is one of the top causes of adult admissions and death, with case fatality rates ranging from 18% to 27% in the initial 30 days post‐stroke [9]. However, the country lacks a comprehensive national stroke strategy, and rehabilitation services remain severely underdeveloped, especially in rural and underserved regions [10]. The absence of national stroke care protocols exacerbates the situation, leaving many stroke survivors with limited access to timely rehabilitation and support services after discharge [11].

The burden of post‐stroke limitations in ADL refers to health or physical problems that impede the ability to perform tasks across all areas of life, ranging from hygiene and hobbies to errands and sleep [12]. These limitations include basic and instrumental daily living activities, including bathing, eating, dressing, toileting, controlling bladder and bowel functions, mobility, transfers, climbing stairs, washing clothes, shopping, and housekeeping [13] Following musculoskeletal and mental issues, stroke is the third leading cause of disability [14], and is frequently the most prevalent cause of complex impairments [15]. Stroke survivors often face mild, moderate, or severe limitations that impact essential life skills such as eating, dressing, getting in and out of bed, using the restroom, moving about, and bathing. Prompt treatment is essential for improving the quality of life for survivors [12].

Maintaining independence in ADL is crucial for quality of life. Stroke survivors who require assistance often experience feelings of social isolation, overwhelm, and abandonment [16]. Among the 50 million stroke survivors worldwide, 25%–74% rely on caregivers for their ADL post‐stroke [17, 18]. This dependency not only burdens family caregivers but also impacts family relationships [19].

Stroke represents a costly illness with significant financial implications globally [14]. Expenses associated with stroke include sedentariness, nursing home care, physician and other healthcare provider services, home healthcare, medications, medical durables, and lost productivity due to illness or death [14]. Moreover, significant financial losses due to medical expenses are another repercussion of limitations [20]. However, the dependence of stroke patients on ADL can be mitigated. Therefore, there is an urgent need to explore ADL factors and develop effective strategies to support stroke recovery.

Several studies have examined factors influencing the prognosis of recovery and ADL limitation in post‐stroke survivors [21, 22, 23]. These factors include a history of sedentariness, the severity of the brain injury, cognitive impairments, access to rehabilitation services, education, smoking habits, post‐stroke depression, lack of early physiotherapy follow‐up, socioeconomic status, age, gender, and living arrangements [11, 22, 23, 24, 25, 26].

In Ethiopia, identifying factors contributing to limitations in ADL among stroke survivors remains limited. Research in the country has predominantly focused on stroke mortality and short‐term recovery, with minimal emphasis on long‐term outcomes like ADL limitations [27]. Previous research on post‐stroke ADL limitations has generally included stroke patients as a whole, without distinguishing between those who experience significant limitations and those who do not [24, 28]. As a result, there is a gap in understanding the specific factors influencing ADL outcomes in this context.

This study addresses that gap by examining the factors associated with ADL limitations among stroke survivors in public tertiary hospitals in the Amhara Regional State of Ethiopia. Its novelty lies in focusing on the unique, under‐researched challenges faced by this population, aiming to provide context‐specific insights that can inform stroke care and rehabilitation strategies in resource‐limited settings.

## 2 Materials and Methods

### 2.1 Study Period and Setting

The study was conducted from April 1 to June 30, 2022, involving 424 participants selected through systematic random sampling from four public tertiary hospitals in the Amhara Regional State of Ethiopia. Among the eight referral public hospitals in the region, these four serve as tertiary care centers. The participants included stroke survivors who were experiencing either their first‐ever stroke or recurrent strokes and were attending outpatient follow‐up care, such as in the medicine or physiotherapy departments, rather than being hospitalized. Both acute stroke survivors and chronic stroke survivors were included in the study to evaluate post‐stroke limitations in ADLs at various stages of recovery.

The inclusion criteria were as follows: stroke survivors aged 18 and older who had either their first or recurrent stroke. Participants had to have a post‐stroke duration of at least 2 weeks and be able to remain conscious and respond to questions. At the time of the study, participants were not hospitalized but were receiving follow‐up outpatient services, such as medication management or physiotherapy.

Patients were excluded if they showed signs of aphasia (communication difficulties preventing meaningful interaction), cognitive impairment, which was assessed by using the Mini‐Mental State Examination checklist [29], or if they refused to participate.

### 2.2 Sample Size Determination, Sampling Technique, and Procedure

The sample size was determined by using EpiData version 4.6.02 and the single population proportion formula, with the following assumptions: a 5% margin of error (*d*), a 95% confidence interval (alpha, *α* = 0.05, two‐tailed), and an estimated population of 792 stroke survivors attending outpatient services during the study period. This estimate of 792 participants, based on the total population of stroke survivors at four selected tertiary hospitals, represented the population from which the sample would be drawn. Additionally, a 50% prevalence for post‐stroke limitations in basic ADLs was assumed, as no prior studies in the region with the same population were available. The 50% prevalence was chosen because it is a standard approach when the true prevalence is unknown, as it provides the maximum sample size estimate and ensures the most conservative and reliable results. This assumption was made due to the lack of prior studies in the region with the same population [30].

Once the sample size was calculated, participants were proportionally distributed across the four public tertiary hospitals based on the population size at each institution involved in the study. A systematic random sampling method was used to select participants from each hospital. Each hospital received a proportionate share of the required participants. The sampling interval (*K* = *N*/n) was calculated, with a value of 2, to ensure that every second stroke survivor was selected for the study.

Participants were selected from stroke survivors either waiting for services or organized sequentially upon arrival at each hospital. To minimize bias, the first participant in each session was selected using a lottery method. Special markers on participant charts and verbal confirmations with participants were employed to ensure data integrity and prevent duplication.

**Figure 1:**
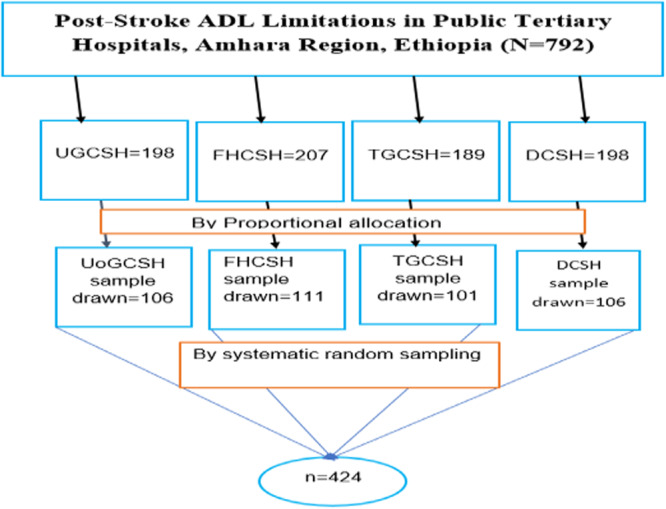
Schematic presentation of sampling procedure among stroke survivors at Amhara Regional Comprehensive Specialized Hospital, northwest of Ethiopia.

The total populations from each hospital were as follows: FHCSH = 207, TGCSH = 189, UOGCSH = 198, and DCSH = 198. The process continued until the required sample size of 424 participants was achieved, with participants selected from each hospital's outpatient department for medicine and physiotherapy (Figure 1).

### 2.3 Data Collection and Procedures

A semi‐structured questionnaire was adapted from previously validated studies [21, 22, 23, 24, 25] to collect data on participants' sociodemographic profiles, clinical characteristics, behavioral factors, and post‐stroke limitations in basic ADLs. The sociodemographic section captured variables such as age, gender, educational status, marital status, occupation, place of residence, and economic status.

Clinical information, including stroke type (ischemic or hemorrhagic), affected cerebral hemisphere (left or right), duration since the stroke, physiotherapy treatment sessions, and type of mobility aids used. To address potential concerns regarding the accuracy of self‐reported clinical information, data regarding stroke type and side were primarily extracted from medical records and verified through chart review. Where necessary, this information was clarified through interviews with participants or caregivers to ensure accuracy and completeness. Behavioral characteristics such as balance impairment, mobility speed, and depression symptoms were also included. To collect the data, the questionnaire was first translated from English to Amharic. Later, it was translated back into English for consistency and further analysis. Data was collected through face‐to‐face interviews, chart reviews, physical examinations, and observations by eight qualified BSc physiotherapists.

### 2.4 The Bartle Index (BI)

BI was used as an outcome tool to measure post‐stroke limitations in basic ADL [31], which assessed 10 items: personal toilet use, getting on and off the toilet, ascending and descending stairs, dressing, bowel and bladder control, feeding, transferring from wheelchair to bed and back, bathing, and walking on a level surface. Scores were calculated by adding the individual scores of each item, ranging from 0 (completely dependent) to 100 (completely independent). For this study, ADL scores on the BI were dichotomized, with “0–60” indicating “limited in basic ADL” and “61–100” indicating “not limited in basic ADL” [31]. The Geriatric Depression Scale–Short Form (GDS‐SF) was utilized to screen for depressive symptoms. This scale comprises 15 items, derived from the original 30‐item GDS. In this study, a GDS score greater than 10 was categorized as “depressed,” while scores of 10 or less were considered “not depressed” [32]. The GDS is a reliable and valid measure that has been confirmed with large samples of post‐stroke patients [33, 34]. The Berg Balance Scale was employed to assess balance impairment [35].

### 2.5 Data Quality Assurance

To ensure data quality, data collectors and supervisors underwent 2 days of training on how to approach study participants and administer the questionnaire. Additionally, the principal investigator provided on‐site supervision. The questionnaire was pre‐tested on 5% of the total sample at Debre Tabor University Referral Hospital to verify response accuracy, language clarity, and tool appropriateness before the actual data collection commenced. The principal investigator and supervisors rigorously reviewed the questionnaire for completeness, accuracy, and clarity prior to data entry. Furthermore, a data cross‐check was performed before analysis to ensure data integrity.

### 2.6 Statistical Analysis

After revision, coding, and cleaning, the collected data were entered into EpiData version 4.6.02. It was then exported to Stata Version 16.0 for analysis. The research population was characterized using descriptive statistics, including percentages, frequencies, means, medians, ranges, and standard deviations for relevant continuous variables. The findings were presented through text, tables, and figures. The variance inflation factor was employed to assess multicollinearity. In logistic regression analyses, variables with a *p*‐value below 0.25 were considered for inclusion [36]. The Hosmer–Lemeshow goodness‐of‐fit test was used to assess the fit of the logistic regression model; variables that did not fit were excluded from the multivariate logistic analysis [37].

Binary logistic regression was utilized to identify sociodemographic, clinical, rehabilitation‐related, mobility‐related, and psychosocial factors independently associated with ADLs. An independent variable that demonstrated a significant association with the dependent variable in bivariate logistic regression at a *p*‐value of less than 0.25 was considered a candidate for the final logistic regression model. These candidates were then subjected to multivariate analysis to control for confounding effects and to identify statistically significant determinants of ADL limitations in stroke survivors. Statistical significance was determined at an AOR with a 95% confidence interval and a *p*‐value of less than 0.05.

### 2.7 Ethical Statement

Ethical approval was obtained from the University of Gondar, School of Medicine Ethical Review Board. The Department of Physiotherapy and the university received letters of support and cooperation.

Participation in this study was entirely voluntary. Participants were fully informed of the purpose, duration, benefits, and potential risks involved, and could choose to decline or withdraw at any time. Data confidentiality was maintained throughout the study, and the information collected was used solely for the intended research purposes. Written consent was obtained from each participant prior to data collection. Participants identified with ADL functional disorders during the study were provided with appropriate advice and referred for further care and management.

## 3 Results

### 3.1 Sociodemographic Characteristics of Participants

The study initially included 424 stroke survivors. However, due to missing data and participant dropout, the final sample size was reduced to 400 stroke survivors. The response rate for the study fell within a 95% confidence interval of 46.58%–56.38%. Participants ranged in age from 18 to 95 years, with 57.5% being female. A majority, 54%, were unmarried. Approximately 48.5% of the participants earned a monthly income of less than 1000 Ethiopian birr, and the largest employment group, 23.25%, worked for non‐governmental organizations (Table 1).

**Table 1:** Sociodemographic features of study participants with the presence and absence of limitations in basic ADL, including *p* value (*n* = 400).

			Limitations in basic ADL			
Variable	Frequency (*n*)	Percent (%)	Yes	No	COR (95% CI)	*p* value
Age						
18–40	101	25.25%	40	61	1	1
41–95	299	74.75%	193	106	2.77 (1.75, 4.42)	0.00
Sex						
Male	170	42.5%	96	74	1 1	1
Female	230	57.5%	137	93	1.14 (0.75, 1.69)	0. 54
Marital status						
Unmarried	216	54%	127	89	1.05 (0.70, 1.56)	0. 81
Married	184	46%	106	78	1	1
Education						
Formal educates	70	17.5%	44	26	1	1
Non‐educate	330	82.5%	189	141	0.79 (0.46, 1.34)	0.39
Residence						
Urbane	179	44.75%	107	72	1	1
Rural	221	55.25%	126	95	0.89 (0.59, 1.33)	0.577
Income level						
< 1000	194	48.5%	114	80	1	1
1001–2000	54	13.5%	30	24	0.877 (0.47, 1.61)	0.67
2001–3000	51	12.75%	30	21	1.00 (0.53, 1.87)	0.99
> 30000	101	25.25%	59	42	0.98 (0.60, 1.60)	0.95

#### 3.1.1 Clinical Characteristics of Study Participants

About half of the participants, 49.75%, had hemorrhagic strokes, while 48.75% experienced ischemic strokes. Additionally, 57% of participants were in the acute post‐stroke phase, and 53.25% did not adhere to their physiotherapy treatment regimens. Furthermore, 59.75% of participants with aids (Table 2).

**Table 2:** Clinical characteristics, with the presence and absence of limitations in basic ADL, including *p*‐value (*n* = 400).

Variables	Frequency (*n*)	Percent (%)	Limitations in basic ADL		COR (95% CI)	*p* value
			Yes	No		
Type of stroke						
Ischemic	195	48.75%	109	86	0.25 (0.03, 2.21)	0.24
Hemorogic	199	49.75%	119	80	0.29 (0.03, 2.59)	0.23
Undetermined	6	1.5%	5	1	1	1
Type of hemisphere						
Right	199	49.75%	109	90	0.02 (0.04, 1.01)	0.053
Left	188	47.00%	113	75	0.27 (0.05, 1.27)	0.10
Undetermined	13	3.25%	11	2	1	1
Post‐stroke duration						
Acute/sub‐acute	228	57%	165	63	4.01 (2.62, 6.10)	0.000
Chronic	172	43%	68	104	1	1
Physiotherapy treatment						
Treated	187	46.75%	65	122	1	1
Not treated	213	53.25%	168	45	7.0 (4.48, 10.94)	0.000
Walking aids						
With aids	239	59.75%	180	59	6.21 (3.99, 9.66)	0.000
Without aids	161	40.25%	53	108	1	1
Comorbidity						
Yes	68	17%	49	19	2.07 (1.17, 3.67)	0.012
No	332	83%	184	148	1	1

#### 3.1.2 Behavioral Characteristics of the Participants

The majority of the participants (54.75%) exhibited abnormal mobility speed. About 53% displayed normal psychosocial behavior or lacked depressive features, and 51.5% reported having balance problems (Table 3).

**Table 3:** Participants' depression, balance impairment, and mobility speed, with the presence and absence of limitations in basic ADLs (*n* = 400).

Variable	Frequency (*n*)	Percent (%)	Limitations in basic ADL		COR (95% CI)	*p* value
			Yes	No		
Depression						
Depressed	188	47.00%	158	30	9.62 (5.94, 15.56)	0.000
Normal	212	53.00%	75	137	1	1
Balance impairment						
Normal	194	48. 5%	71	123	1	1
Impaired	206	51.5%	162	44	6.378 (4.09, 9.93)	0.000
Speed of mobility						
Abnormal	219	54.75%	167	52	5.59 (3.62, 8.63)	0.000
Normal	181	45.25%	66	115	1	1

The factors included in the bivariate logistic regression analysis were age, use of walking aids, mobility speed, morbidity, type of stroke, affected hemisphere, duration since stroke, balance impairment, physiotherapy treatment adherence, and presence of depression. Several of these variables showed *p*‐values less than 0.25, indicating they are suitable candidates for inclusion in the final logistic regression (Table 4).

**Table 4:** Multivariate logistic regression of factors linked to limitations in basic ADLs among stroke survivors (*n* = 400).

	Limitations in basic ADL				
Variable	Yes	No	COR (95% CI)	AOR (95% CI)	*p* value
Age	40	61	1	1	1
18–40					
41–95	193	106	2.77 (1.75, 4.42)	0.35 (0.22, 0.92)	0.225
Depression					
Yes	158	30	9.62 (5.94, 15.56)	1.69 (0.88, 2.49)**	**0.00**
No	75	137	1	1	1
BI					
No	71	123	1	1	1
Yes	162	44	6.378 (4.09, 9.93)	0.19 (1.08, 4.70)	0.672
Stroke duration					
Acute/sub‐acute	165	63	4.01 (2.62, 6.10)	0.18 (0.84, 1.77)	0.586
Chronic	68	104	1	1	1
Physiotherapy					
Not treated	168	45	7.0 (4.48, 10.94)	1.88 (0.19, 1.58)*	**0.013**
Treated	65	122	1	1	1
Walking aids					
With aids	180	59	6.21 (3.99, 9.66)	1.81 (0.17, 1.44)*	**0.012**
Without aids	53	108	1	1	1
Speed of mobility					
Abnormal	167	52	5.59 (3.62, 8.63)	0.23 (1.03, 2.57)	0.571
Normal	66	115	1	1	1
Comorbidity					
Yes	49	19	2.07 (1.17, 3.67)	0.13 (0.55, 0.83)	0.695
No	184	148	1	1	1
Hemisphere					
Right	109	90	0.02 (0.04, 1.01)	1.79 (3.58, 5.010)*	**0.049**
Left	113	75	0.27 (0.05, 1.27)	1.39 (3.18, 4.39)	0.126
Undetermined	11	2	1	1	1
Stroke type					
Ischemic	109	86	0.25 (0.03, 2.21)	1.39 (3.85, 7.21)	0.306
Hemorogic	119	80	0.29 (0.03, 2.59)	1.21 (3.72, 1.29)	0.342
Undetermined	5	1	1	1	1

*Note:* Bold indicates the significant explanatory variables with their COR, AOR, and CI, 1 = reference category; *Significant at *p* < 0.05 and **Significant at *p* < 0.001.

Abbreviations: ADL, activity of daily living; AOR, adjusted odds ratio; BI, balance impairment; CI, confidence interval; COR, crude odds ratio.

## 4 Discussion

Post‐stroke limitations in ADLs remain a significant global health concern, with prevalence and contributing factors varying by region due to differences in socioeconomic status and healthcare infrastructure [38]. High‐income countries typically report lower prevalence rates of post‐stroke limitations, often attributed to early stroke detection, advanced acute management, and comprehensive rehabilitation services [39]. In contrast, LMICs, particularly in Africa, continue to report higher rates of post‐stroke limitations, largely due to systemic healthcare challenges and socioeconomic constraints [40].

In our study, the prevalence of limitations in basic ADL among stroke survivors was 58.25%. This figure reflects a moderate‐to‐high burden of disability. This finding is consistent with results reported from other LMICs, particularly within SSA. Importantly, the study found a high prevalence of activity limitations among chronic stroke survivors in Benin, a setting that shares similar sociocultural and economic characteristics with ours, underscoring the persistent difficulties faced by stroke survivors in this region [41]. Outside the African context, our prevalence estimate aligns with findings from studies conducted in China [38], Northeastern India [37], India (37.4%) [39], and Tunisia [38], possibly due to similar operational definitions, methodologies, and participant characteristics such as age. Like our study, those conducted in China and India employed the Barthel Index for assessing post‐stroke limitations in basic ADL. The Tunisian study's institution‐based setting and participants' educational profile also closely mirror our own demographics.

In contrast, lower prevalence rates have been reported in Brazil [42] and Lebanon [43], potentially due to methodological differences, such as the use of the Katz Index (less sensitive than the Barthel Index), or contextual factors such as higher baseline physical activity among rural Lebanese residents. Conversely, our prevalence was lower than that reported in studies from Iran and Canada [44, 45], which may be attributed to older or institutionalized populations with more severe impairments, or differences in inclusion criteria.

In examining factors associated with basic ADL limitations, our study identified several key predictors that are also reflected in the broader literature. Stroke survivors who did not follow physiotherapy treatment were 1.88 times more likely to experience limitations, which was consistent with findings from studies in Australia, the Netherlands [46, 47], and SSA, including the Benin study [41]. Limited access to physiotherapy is a recurrent barrier across many African settings, contributing significantly to poor functional outcomes post‐stroke. The likely explanation is that regular exercise plays a crucial role in preventing post‐stroke limitations by improving muscle function, range of motion, balance, lean muscle mass, and coordination. Furthermore, exercise can prevent or delay the onset of chronic conditions, increase bone density, and enhance social interactions, which may prevent symptoms of depression [48].

Depressive symptoms were also significantly associated with limitations in basic ADL in our study. Stroke survivors with symptoms of depression were 1.69 times more likely to be functionally limited in ADL, mirroring findings from the Benin study and other global literature [36, 41, 49, 50, 51, 52]. Depression not only reduces motivation and participation in rehabilitation but also affects physical performance and recovery. This underscores the importance of integrating mental health services into stroke rehabilitation, particularly in SSA countries where such services are often lacking or stigmatized.

The study found that stroke survivors using mobility aids (walking aids) were 1.81 times more likely to develop limitations in basic ADL compared to those who did not use mobility aids. This finding aligns with several previous studies [53, 54, 55]. It is possible that using mobility aids increases the cognitive and attentional demands of tasks, reducing intervention efficiency [56]. This might indicate that users of walking aids are often engaged in dual‐tasking, which involves performing multiple functional tasks in basic ADL simultaneously.

Furthermore, the right hemisphere involvement in stroke survivors was associated with post‐stroke limitations in basic ADL. In contrast, some studies did not find the right hemisphere to be a significant factor in post‐stroke limitations in basic ADL [57, 58]. This discrepancy may be due to differences in the participant populations. In the current study, we recruited survivors of a first‐ever stroke who had right hemisphere involvement and were limited, unlike the studies in references [57, 58], which considered different participant criteria. Therefore, further research is needed to explore the relationship between right hemisphere involvement and post‐stroke limitations in basic ADL.

## 5 Limitations of the Study

This study has several limitations. Key variables potentially associated with post‐stroke limitations in basic ADL, such as stroke severity, nutritional status, aphasia, cognitive impairment, and mental illness, were not included. Individuals who did not attend the public tertiary hospital during the data collection period were excluded, which might affect the generalizability of the findings. The sample was drawn from a single regional state tertiary public health facility; thus, the characteristics of the broader population might not be accurately represented, given that stroke management varies across different facilities. Although using the Barthel Index was time‐efficient, it does not assess post‐stroke limitations in instrumental ADLs, which require more complex functions, such as cognitive processing. Additionally, other potential impairments, such as pain, loss of sensation, spasticity, and decreased coordination, that could affect participants' ability to perform ADLs were not considered. Due to its cross‐sectional design, the study cannot establish causation. Future studies should involve larger samples of stroke survivors, assessing both basic and instrumental ADLs across various degrees of post‐stroke limitation.

## 6 Conclusion

Our results significantly contribute to the body of knowledge regarding post‐stroke limitations in basic ADL among stroke survivors. According to our findings, 58.25% of stroke survivors at public tertiary hospitals exhibited limitations in basic ADL. The results also demonstrate a strong association between post‐stroke limitations in basic ADL and several factors: symptoms of depression, lack of physiotherapy treatment, involvement of the right hemisphere, and the use of mobility aids. These limitations may adversely impact the daily activities, social interactions, and work performance of stroke survivors.

## Author Contributions

**Melese Gobezie:** conceptualization, methodology, software, data curation, formal analysis, project administration, funding acquisition, investigation. **Getachew Azeze Eriku:** funding acquisition, writing – original draft, validation, formal analysis, supervision. **Tesfa Kassa:** writing – original draft, writing – review and editing. **Destaw Marie Merawie:** writing – original draft, validation, writing – review and editing, resources. **Samual Mersha Birru:** funding acquisition, visualization, software, data curation, resources. **Setegn Fentahun:** investigation, validation, formal analysis, supervision. **Gerum Nakie:** funding acquisition, validation, project administration, resources. **Jemal Suliuman:** investigation, funding acquisition, visualization, formal analysis, resources. **Mulualem Kelebie:** conceptualization, methodology, software, data curation. **Alemu Kassaw Kibret:** investigation, validation, formal analysis, project administration, supervision.

## Funding

The authors received no specific funding for this work.

## Conflicts of Interest

The authors declare no conflicts of interest.

## Transparency Statement

The lead author, Melese Gobezie, affirms that this manuscript is an honest, accurate, and transparent account of the study being reported; that no important aspects of the study have been omitted; and that any discrepancies from the study as planned (and, if relevant, registered) have been explained.

## Data Availability

The dataset supporting the conclusions of this article is available via the principal investigator upon reasonable request.
